# Estimating the Loss in Expectation of Life and Relative Survival Rate among Hemodialysis Patients in Iran

**DOI:** 10.34172/jrhs.2020.21

**Published:** 2020-08-03

**Authors:** Navisa Sadat Seyedghasemi, Abbas Bahrampour, Abbas Etminan, AliAkbar Haghdoost, Mohammad Reza Baneshi

**Affiliations:** ^1^Modeling in Health Research Center, Institute for Futures Studies in Health, Kerman University of Medical Sciences, Kerman, Iran; ^2^Department of Biostatistics and Epidemiology, Kerman University of Medical Sciences, Kerman, Iran; ^3^Adjunct Professor of Griffith University, Brisbane, QLD, Australia; ^4^Physiology Research Center, Departments of Nephrology, Urology and Renal Transplantation, Kerman University of Medical Sciences, Kerman, Iran; ^5^HIV/STI Surveillance Research Center, and WHO Collaborating Center for HIV Surveillance, Institute for Futures Studies in Health, Kerman University of Medical Sciences, Kerman, Iran

**Keywords:** Life Expectancy, Hemodialysis, Survival, Prognosis

## Abstract

**Background:** Information regarding the prognosis and burden of diseases can be used by policymakers to determine competing health priorities. We aimed to assess the Relative Survival Rate (RSR) and loss of expectation of life (LEL) to evaluate the prognosis and burden of diseases in Hemodialysis (HD) patients.

**Study design:** A retrospective cohort study.

**Methods:** We recruited 648 HD patients referred to three referral centers in Kerman City, Iran, from 2008 to 2019. RSR, was defined as the ratio of the observed and the expected survival rates of general population for persons of the same age and sex as patients in the current study. LEL was determined as the difference between corresponding life expectancies (LE). The extended Cox proportional hazard model was used to identify variables associated with the outcome.

**Results:** Variables associated with outcome were diabetic status and age. In the 5th year of the follow-up study, the overall RSR was 0.57. In general, for HD patients, the estimation of LE and LEL was 22.6 and 12.36 year, respectively.

**Conclusion:** HD patients, especially older patients, showed a very poor prognosis, with a large amount of lost life expectancy. Therefore, they need more care and attention from health authorities. It is suggested to estimate the cost of eliminating the risk factors causing kidney diseases.

## Introduction

End-stage renal disease (ESRD) is the worst stage of chronic kidney disease (CKD). A person with ESRD will need to have treatment to replace a damaged kidney to stay alive. Renal replacement therapy (RRT) includes hemodialysis (HD), peritoneal dialysis (PD), and kidney transplantation^1,2^. ESRD causes disturbance in life (perhaps even more than any other chronic illnesses) mainly due to severe metabolic and cardiovascular complications ^3^. The growth of ESRD is a worldwide challenge ^4^. In Iran, the annual number of patients with ESRD increased by 130% between 2000 and 2006^5,6^. Based on the data available in the 2018 annual report accessible on the US Renal Data System, the crude incidence rate of treated ESRD and the prevalence of treated ESRD were 81 per million probabilities/year and 654 per million population, respectively, in Iran ^7^. Using 1995-2013 statistics, the overall trend of survival probabilities in ESRD patients has slightly decreased in Iran ^8^. About half of the patients with ESRD in Iran are treated with hemodialysis^2^.

Survival statistics (e.g., Cause-specific survival and overall survival) as well as loss of expectation of life (LEL) were used to provide information regarding the prognosis and burden of diseases. This information can be used by policymakers to determine competing health priorities ^9^.

Cause-specific (or net) survival represents the survival associated with a specific cause. The main limitation of this method is that it relies on reliable causes of mortality. ESRD rarely is considered a cause of death; rather, it commonly is considered as a factor that enhances the effect of other causes, such as infections and heart diseases.

On the other hand, the RS method compares the overall survival of the patients with that of the general population. It matches the two populations by key variables such as age and sex. In the RS method, the overall survival rate of patients (i.e. when all deaths are considered events) is compared with the expected survival rate (in the absence of that specified disease)^9-11^. In conditions where estimating the cause-specific survival is not possible, the relative survival (RS), which requires no further information regarding the cause of death, is a preferable method^9,10^.

Another useful statistic to compare survival experience of patients with that of the general population is LEL, defined as the difference between life expectancy (LE) of patients and expected value in general population^12,13^. Both expected survival rate and anticipated LE can usually be obtained from national or regional life tables ^9-11^.

In our extensive search, we could not find any information regarding the RSR and LEL of Iranian ESRD patients. We aimed to determine the variables associated with survival of ESRD patients, and to provide an estimate of their RSR and LEL statistics.

## Methods

In this retrospective study, we enrolled 801 HD patients from Mar 2008 to Jan 2019 in Kerman City, the capital of the largest province located in the southeastern part of Iran. Kerman is the 9^th^ most populated province of Iran and covers more than 11% of its land ^14^. Since Kerman is close to the country average, in terms of healthcare and human development indicators, it can be considered as a sample representing the entire country^15-17^.

Data were extracted from the patients’ records at three referent hemodialysis centers. The exclusion criteria were as follows: (1) individuals less than 18 yr;) 2) individuals who died in the first three months after dialysis treatment starts; and )3) patients with incomplete information on age, sex, and starting date of dialysis.

The main outcome of this study was death. For cases who died, the follow-up time was defined as the difference between date hemodialysis started and death date. For censored cases, difference between starting hemodialysis date and last observation date was calculated (i.e. Jan 6^th^, 2019). The independent variables were main causes of ESRD, sex, blood group, diabetic status, and age at the beginning of hemodialysis.

In terms of age, patients were categorized into six groups: 18-34 yr: 35-44 yr: 45–54 yr: 55-64 yr: and ≥65 yr. The primary cause of ESRD was determined by ICD-10-CM diagnosis codes. As diabetes is a significant matter that affects the survival rate of HD patients, patients were placed into two groups of diabetics and non-diabetics from the very beginning of their hemodialysis.

A Cox Proportional Hazard (PH) model was developed to investigate the association between independent variables and the outcome. PH assumption was checked using interaction with time terms to the model and Shoenfeld residuals. In the case that data did not satisfy the PH assumption, results of the extended Cox model were reported.

To calculate the RSR, patients were matched with general population in terms of age, gender, and year of diagnosis. The relative survival rate (RSR) was calculated as the overall (or all-cause) survival rate of HD patients (shown by S(t)), divided by the expected survival rate (shown by S*(t)):

(1) $$RSR(t)=\frac{S(t)}{S*(t)}$$

We obtained S(t) using Kaplan-Meier method. S*t was estimated using Hakulinen method^18^.

The difference between RSR(t) and S(t) showed the fraction of the death rate due to other causes than hemodialysis^19^.

LE over (0, t) period was calculated as t- mean survival time. The mean survival time was calculated as the area under the K-M survival curve. LEL was calculated as the difference between expected life expectancy of general population (LE*) and that of patients (LE)^20^:

(2) LEL=LE*−LE

Applying Hakama and Hakulinen method^18,20^, S*t and LE* were obtained by linking HD patients by sex, age, and year to the Kerman province (2008–2019) life tables. Adopting the methodology provided by the World Human Mortality Database, life table of general population has been prepared by the first author of this manuscript.

This method requires two types of data including mortality and general population statistics gathered from two sources: the death registry of Health Deputy of Kerman University of Medical Sciences, and the census data of the Statistical Centre of Iran, respectively.

All analyses were performed in the R Software, Release 3.5.3, by using survival and survMisc packages.

## Results

Of the 801 patients, 153 cases were excluded as they did not meet the inclusion criteria, and the sample analysis was set at 648 cases. During the study, 54 (8.3%) patients received a renal transplant.

We classified patients based on their cause of ESRD. About 80% of patients had a history of either diabetes or hypertension: Diabetes and hypertension (30.7%), diabetes alone (27.2%), and hypertension alone (23%).

The mean age at the start of hemodialysis treatment was 58.29 ±15.011 (58.7m±15.3 for males and 57.6 ±14.6 for females) (Table 1).

**Table 1:** Relative survival rate, Life expectancy, and loss in expectation of life for patients who began hemodialysis between March 2008 and January 2019 in Kerman City, Iran

**Variables**	**Age (yr)** **mean ±SD**	**5-year relative** **survival rate (95% CI)**	**Deaths** ^a^	**Mean of life expectancy**		**Expectation of life lost** **for hemodialysis patients**
				**General** **population**	**Hemodialysis** **patients (95% CI)**	
Age (yr)						
18-35	28.4 ±4.4	0.76 (0.58, 0.93)	0.3	61.1	45.87 (36.6, 55.1)	15.26
36-45	41.2 ±2.8	0.69 (0.47, 0.92)	0.5	50.2	35.58 (25.4, 45.8)	14.63
46-55	51.1 ±2.7	0.63 (0.48, 0.78)	1.0	40.2	25.78 (19.9, 31.7)	14.38
56-65	60.4 ±2.8	0.59 (0.48, 0.72)	2.1	32.0	20.05 (16.8, 23.3)	11.98
≥66	73 ±6.0	0.45 (0.33, 0.57)	4.8	23.2	12.20 (10.6, 15.5)	11.01
Sex						
Female	57.6 ±14.6	0.54 (0.43, 0.65)	2.2	35.2	19.61 (16.4, 22.8)	15.56
Male	58.7 ±15.3	0.58 (0.50, 0.66)	3.0	34.8	21.29 (18.9, 23.7)	13.47
Diabetes status						
Diabetics	62.1 ±11.3	0.53 (0.44, 0.62)	2.9	31.6	18.66 (16.0, 21.3)	12.95
Non-diabetics	53.0 ±17.7	0.61 (0.52, 0.70)	2.4	39.5	27.68 (24.2, 31.2)	11.79
Total	58.3 ±15.0	0.57 (0.50, 0.63)	2.7	34.9	22.56 (20.4, 24.7)	12.36

^a^ Percentage of deaths from causes other than those associated with or due to hemodialysis at 5th year of follow up; 5-year RSR minus 5-year survival rates

Patients were mostly male (61%) and the most frequent age group was 55-64 yr (Table 2). The blood group of 20 patients was not registered in hospital records. The most prevalent blood group was O (36.6%) followed by A (28.5%). Only 8.4% of patients had AB blood group (Table 2).

**Table 2:** Baseline characteristics of patient, result of Kaplan Meier analysis, and estimation of model parameters Cox extended with g (t) = t in patients who began hemodialysis between March 2008 to January 2019 in Kerman City, Iran

**Variables**	**n (%)**	**Mean (Median)** **survival time** ^a^	**5-year** **survival rates**	***P*** **value for** **Proportional Hazard** **assumption Test**	**Hazard Ratio** **(95% CI)**	***P*** **value**
Total	648 (100)	6.4 (6)	0.54	0.171		
Sex						
Male	395 (61.0)	6.6(6)	0.55	-	1.00	
Female	253 (39.0)	5.8 (5)	0.52	0.080	1.09 (0.83, 1.43)	0.557
Age group (yr)						
18-34	69 (10.6)	6.5 (-)	0.76	-	1.00	
35-44	56 (8.6)	6.5 (-)	0.69	0.278	1.23 (0.53, 2.85)	0.627
45-54	104 (16.0)	6 (-)	0.62	0.581	1.75 (0.86, 3.57)	0.123
55-64	192 (29.6)	5.7 (6)	0.57	0.549	2.16 (1.12, 4.18)	0.021
≥65	227 (35.2)	4.4 (4)	0.40	0.425	3.24 (1.71, 6.17)	0.001
Diabetes status						
Non-diabetics	273 (42.1)	7.03 (-)	0.59	-	1.00	
Diabetics	375 (57.9)	5.74 (5)	0.50	0.027	0.6 (0.38, 0.95)	0.029
Diabetes status×t	-	-	-	-	1.3 (1.08, 1.54)	0.005
Blood group ^a^						
O	230 (36.6)	6.3 (6)	0.52	-	1.00	
A	179 (28.5)	7.2 (-)	0.57	0.380	0.89 (0.62, 1.27)	0.524
B	166 (26.4)	6.0 (5)	0.43	0.788	1.33 (0.95, 1.85)	0.094
AB	53 (8.4)	5.1 (4)	0.34	0.534	1.36 (0.85, 2.16)	0.195

^a^ Median for subgroups whose survival rate was no less than 0.5, it is impossible to calculate the median survival time.

Total person-year at risk was 1775. During the study period, 234 patients died (36.1 %) which gave a crude mortality rate of 13.18 deaths/100 patient-years (95% CI: 12.02, 15.55).

**Figure 1:**
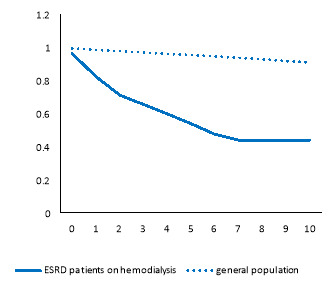
Survival rate curve observed in ESRD patients on hemodialysis versus the survival rate curve of the general population

By comparing the survival rate of ESDR patients with that of the general population, patients experienced poor survival rates (Figure 1). The percentage point difference between the survival rate of the general population and that of the patients at 1th and 5th years was 16% (0.98 vs. 0.82) and 41.4% (0.95 vs. 0.54), respectively (Table 1). Only 3% of deaths (57% minus 54%) that occurred during the first 5 years of hemodialysis were due to causes other than hemodialysis. Stratifying the analysis by age, corresponding figures for those aged between 18 to 35 and those aged more than 65 were 0.3% and 4.8% respectively (Table 1).

**Figure 2:**
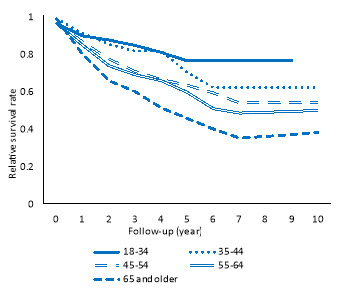
Relative survival rate by age category

Figure 2 suggests that in all age groups, patients had poorer survival than their counterparts in the general population. Moreover, the older the age, the worsen RS.

**Figure 3:**
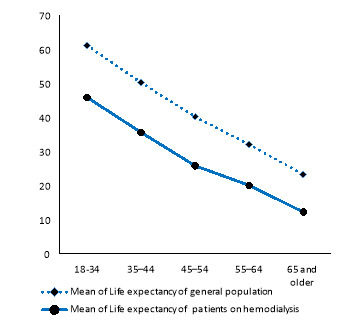
Comparison of LE in HD patients and their respective reference populations by age groups

The LE and LEL for patients were 22.56 and 12.36 yr, respectively. Subgroup analysis revealed that LEL in the youngest and oldest age groups was 15.26 and 11 yr, respectively (Table 1, Figure 3). As demonstrated in Table 2, LEL for females was greater than that of males (13.5 vs. 15.6 years). Moreover, LEL for diabetics and non-diabetics were 12.9 and 11.8 yr, respectively.

The median survival time was 6 years. Moreover, 5-yr KM survival rate was 54.0% (55% in males and 52% in females). As the participants’ age increased, the 5-year survival rate decreased. The percentage point difference, in terms of 5-year KM survival, between blood groups A and AB was as high as 0.23 (0.57 versus 0.34). The corresponding figure between diabetic and non-diabetic patients was 0.09 (0.50 versus 0.59) (Table 2).

The only variable that does not satisfy the PH assumption was the diabetic status (Table 2, *P* =0.030). The Kaplan–Meier curves (not shown in the results) showed that the survival rate of diabetics and non-diabetic patients were more or less the same in the first five years of hemodialysis. However, after the 5th year, non-diabetic patients had a higher survival rate. To assess the impact of this variable on the outcome, an interaction term between diabetes status and time was added in the multifactorial Cox model.

Relative to those aged 34 yr or less, individuals whose age was between 55 and 64 were about two times more likely to die (Table 2). Blood groups and sex were not significantly associated with the outcome. Although the hazard of death for diabetic patients was 40% less than non-diabetic cases at the beginning of the study, the hazard rate of death in diabetics relative to non-diabetics increased to 2.13 in the 5th year (95% CI: 1.07, 3.57).

## Discussion

Our results provide important results from policy-making and clinical perspective. At the end of the fifth year of hemodialysis, the patients' survival rate was less than what was expected for the general population by 43.4%. The estimation of LEL revealed that 12 life-years could be gained from successful prevention of causes of ESRD. The highest burden of disease has been observed in females and young patients. Patient with diabetes and patients who are over 65 yr old had the highest risk of death and the worst prognosis of ESRD.

Our data demonstrated that the survival rate during the first five years was 53.89%. Based on previous studies carried out in various areas of Iran, the 5-year survival rate of dialysis patients was calculated at 18.4% in the northern part of Iran, 16% in the western, and 48.6% in the southern ^21-23^. In another study, the five-year survival rate for ESRD patients in the USA, Japan, and Europe is reported to be 41%, 60%, and, 48%, respectively ^2^. The observed differences between the survival rates of ESRD patients in several areas might be due to differences in age distribution of patients and access to health facilities.

The use of arteriovenous fistula in the USA was lower than in Europe and Japan. Furthermore, the survival of dialysis patients in Japan substantially was higher than other parts of the world. They justified that’s findings by fewer number of transplant recipients and lower background general population mortality rates in Japan ^2^.

The overall mortality rate in our study was 13.18 per 100 person-years. This rate is less than that of the Italian Dialysis and Transplantation Registry (IDTR) patients (15.68 per 100 person-years) whose median age was 70 ^24^. Diversity in population age structure might justify part of the observed differences. Patients who participated in IDTR study were older than our sample, and this may partially justify the differences.

As RSR adjusts the survival estimations for important potential confounders like age and sex, it is possible to compare the prognosis of patients in different countries with different population structures. The 5-yr RSR for the cases in our study and the IDTR patients was 56.56% and 55.6%, respectively ^24^, indicating that the prognosis of patients is similar in both societies.

One of the main results of the present study was the reverse association between age and RSR. An increase in age was associated with decrease in RSR. This finding is in line with other studies ^24-29^. It suggests that the prognosis of many diseases is worse in older patients than in younger patients.

In line with another study ^24^, RSR in the fifth year was a little higher in males than in females. According to the life tables proposed by the authors of this manuscript, one reason may perhaps be that the survival rate in the general population of Kerman was higher in females than in males.

According to the calculated percent of deaths from other causes at 5th year of follow up, deaths due to causes other than ESRD were much more frequent in males than in females. A possible explanation for this finding might be that male patients on hemodialysis have a higher prevalence of co-morbidities and other chronic diseases than female patients.

This study indicates that a HD patient could be alive for about 22.6 yr with hemodialysis therapy and lose 12.36 yr of LE on average. This finding is similar to the study conducted in Taiwan^30^. Another study on American adults who received RRT, stated that “LEL decreased from 23.6 yr in 1977 to 19.7 yr in 2007”^31^.

In our study, the higher the age of the patient the less the LEL. This result may reflect that the burden of disease of ESRD in younger patients is more than that in older patients. This trend has also been observed in numerous cancer cases and other diseases ^32-34^. Increase in age was associated with decrease in RSR but decrease in LEL. The same pattern has been reported for cancer patients^11,35,36^. Although seemingly paradoxical, this finding is explained by the higher LE in the younger general population relative to the older general population. In other words, since young patients have more years to lose than old patients, LEL in young patients is greater than that in elderlies.

Similar to other studies, our results showed that female patients lose more years than male patients^24,31,32^. Even though the LE for females is more than that of males in the general population, the LE for female HD patients is less than that of male HD patients. This is an example of reverse epidemiology that may be found in dialysis patients.

In this study, the estimation of the LEL for diabetic HD patients was 13 years. Since diabetics’ life table in the general population was not available, it was not possible to determine how many years of LEL was due to diabetes and how many years of LEL was due to hemodialysis. According to the findings of previous studies, individuals who have diabetes live an average of 6-8 years less than others ^37-39^; thus, it may be inferred that among the diabetic HD patients in the current study, 5 to 7 years of the estimated LEL is due to hemodialysis.

Although the LE for diabetic patients was 9 yr less than the non- diabetic patients, the LEL for diabetics was just one year more than that of non-diabetics. The reason is that the diabetics were older than the non-diabetics at the beginning of hemodialysis; therefore, diabetics have fewer years to lose than non-diabetics (anticipated LE was 31.6 yr in diabetics vs. 39.5 yr in non-diabetics).

According to the result of the multifactorial Cox proportional-hazard model, diabetes status and age variables were important predictors of mortality, as previous studies have shown^23,30,40^.

By using the Kaplan-Meier curves and Cox model, we demonstrated that the survival rate of patients with diabetes had significant differences after the fifth year of hemodialysis compared to patients without diabetes. Beladi Mousavi et al. analyzed the survival of 185 adult HD patients in Ahvaz, Iran. They found that the survival rates of diabetic patients were significantly less than that of non-diabetic patients in the third and fifth years of follow up period ^23^.

Although Kerman is a city whose healthcare and developmental indicators are quite close to the average of that of Iran ^15-17^, generalizability of the results to the whole country should be with caution. Moreover, we have information of a limited number of independent variables. Therefore, information on major independent variables such as socioeconomic status and healthy behaviors on survival remains to be addressed.

Despite these limitations, this study presented beneficial information on patients’ prognosis and the burden of disease through RSR and LEL. To our knowledge, this is the first in Iran to measure the prognosis of HD patients by determining its relationship to the background survival rate of the general population.

## Conclusion

HD patients, especially older patients, showed a very poor prognosis, with a large amount of lost life expectancy. Therefore, they need more care and attention from health authorities. It is suggested to estimate the cost of eliminating the risk factors causing kidney diseases. Additionally, the attempt to estimate the cost of eliminating the risk factors causing kidney diseases would be a valuable effort.

## Acknowledgements

This study was a part of an Ph.D. thesis by Navisa Sadat Seyedghasemi, supported by Kerman University of Medical Sciences and Samen al-Hojaj Special Patients Medical Center, Shafa Hospital, and Javadolaemeh Clinic.

## Conflict of interest

None declared.

## Funding

No funding was received.

## Highlights

- The Hemodialysis patients' survival rate was less than what was expected for the general population by 43.4%.
- The life expectancy of Hemodialysis patients was averagely 12 yr less than that of the general population.
- In Hemodialysis patients, the highest lost life expectancy belonged to females and youth.
- In Hemodialysis patients, patients with diabetes and patients over 65 yr of age had the poorest prognosis.

## References

[1] Najafi I, Hakemi M, Safari S, Atabak S, Sanadgol H, Nouri-Majalan N (2010). The story of continuous ambulatory peritoneal dialysis in Iran. Perit Dial Int.

[2] Robinson BM, Akizawa T, Jager KJ, Kerr PG, Saran R, Pisoni RL (2016). Factors affecting outcomes in patients reaching end-stage kidney disease worldwide: differences in access to renal replacement therapy, modality use, and haemodialysis practices. Lancet.

[3] Weisbord SD, Fried LF, Arnold RM, Fine MJ, Levenson DJ, Peterson RA (2005). Prevalence, severity, and importance of physical and emotional symptoms in chronic hemodialysis patients. J Am Soc Nephrol.

[4] Meguid El Nahas A, Bello AK (2005). Chronic kidney disease: the global challenge. Lancet.

[5] Aghighi M, Mahdavi-Mazdeh M, Zamyadi M, Heidary Rouchi A, Rajolani H, Nourozi S (2009). Changing epidemiology of end-stage renal disease in last 10 years in Iran. Iran J Kidney Dis.

[6] Wetmore JB, Collins AJ (2016). Global challenges posed by the growth of end-stage renal disease. Ren Replace Ther.

[7] Saran R, Robinson B, Abbott K. US Renal Data System 2018 Annual Data Report: Epidemiology of Kidney Disease in the United States. USRDS Web Site; 2019 [updated 1 March 2019; cited 2020]; Available from: https://www.ajkd.org/article/S0272-6386(19)30035-6/pdf. 10.1053/j.ajkd.2019.01.001 PMC6620109 30798791

[8] Heidary Rouchi A, Mansournia MA, Aghighi M, Mahdavi-Mazdeh M (2018). Survival probabilities of end stage renal disease patients on renal replacement therapy in Iran. Nephrology.

[9] Mariotto AB, Noone AM, Howlader N, Cho H, Keel GE, Garshell J (2014). Cancer survival: an overview of measures, uses, and interpretation. J Natl Cancer Inst Monogr.

[10] Ederer F, Axtell LM, Cutler SJ (1961). The relative survival rate: a statistical methodology. Natl Cancer Inst Monogr.

[11] Andersson TM-L, Dickman PW, Eloranta S, Lambe M, Lambert PC (2013). Estimating the loss in expectation of life due to cancer using flexible parametric survival models. Stat Med.

[12] Andersson TML, Rutherford MJ, Lambert PC (2019). Illustration of different modelling assumptions for estimation of loss in expectation of life due to cancer. BMC Med Res Methodol.

[13] Syriopoulou E, Bower H, Andersson TM, Lambert PC, Rutherford MJ (2017). Estimating the impact of a cancer diagnosis on life expectancy by socio-economic group for a range of cancer types in England. Br J Cancer.

[14] Hosseinzadeh J. Population and Housing Censuses. Statistical Center of Iran; 2016 [updated 13 July 2020; cited 2020]; Available from: https://www.amar.org.ir/english/Population-and-Housing-Censuses.

[15] Etemad K, Yavari P, Mehrabi Y, Haghdoost A, Motlagh ME, Kabir MJ (2015). Inequality in Utilization of In-patients Health Services in Iran. Int J Prev Med.

[16] Movahedi M, Hajarizadeh B, Rahimi A, Arshinchi M, Amirhosseini K, Haghdoost AA (2009). Trends and geographical inequalities of the main health indicators for rural Iran. Health Policy Plan.

[17] Noorbakhsh F (2002). Human Development and Regional Disparities in Iran: A Policy Model. J Int Dev.

[18] Hakulinen T (1982). Cancer Survival Corrected for Heterogeneity in Patient Withdrawal. Biometrics.

[19] Glaser N, Persson M, Jackson V, Holzmann MJ, Franco-Cereceda A, Sartipy U (2019). Loss in life expectancy after surgical aortic valve replacement: SWEDEHEART Study. J Am Coll Cardiol.

[20] Hakama M, Hakulinen T (1977). Estimating the expectation of life in cancer survival studies with incomplete follow-up information. J Chronic Dis.

[21] Khazaei S, Yaseri M, Nematollahi S, Zobdeh Z, Sheikh V, Mansournia MA (2018). Survival Rate and Predictors of Mortality among Hemodialysis Patients in West of Iran, 1996-2015. Int J Prev Med.

[22] Montaseri M, Charati JY, Espahbodi F (2016). Application of Parametric Models to a Survival Analysis of Hemodialysis Patients. Nephrourol Mon.

[23] Beladi Mousavi SS, Hayati F, Alemzadeh Ansari MJ, Valavi E, Cheraghian B, Shahbazian H (2010). Survival at 1, 3, and 5 years in diabetic and nondiabetic patients on hemodialysis. Iran J Kidney Dis.

[24] Nordio M, Limido A, Maggiore U, Nichelatti M, Postorino M, Quintaliani G (2012). Survival in patients treated by long-term dialysis compared with the general population. Am J Kidney Dis.

[25] Nelson CP, Lambert PC, Squire IB, Jones DR (2008). Relative survival: what can cardiovascular disease learn from cancer?. Eur Heart J.

[26] Mosavi-Jarrahi A, Abadi A, Mehrabi Y, Mahmoodi M, Eshraghian MR, Mohammad K (2015). Relative Survival of Breast Cancer Patients in Iran. Asian Pac J Cancer Prev.

[27] Davis JS, He V, Anstey NM, Condon JR (2014). Long term outcomes following hospital admission for sepsis using relative survival analysis: a prospective cohort study of 1,092 patients with 5 year follow up. PLoS One.

[28] Pokhrel A, Hakulinen T (2008). How to interpret the relative survival ratios of cancer patients. Eur J Cancer.

[29] Lambert PC, Dickman PW, Nelson CP, Royston P (2010). Estimating the crude probability of death due to cancer and other causes using relative survival models. Stat Med.

[30] Kao TW, Huang JW, Hung KY, Chang YY, Chen PC, Yen CJ (2010). Life expectancy, expected years of life lost and survival of hemodialysis and peritoneal dialysis patients. J Nephrol.

[31] van Walraven C, Manuel DG, Knoll G (2014). Survival trends in ESRD patients compared with the general population in the United States. Am J Kidney Dis.

[32] Neild GH (2017). Life expectancy with chronic kidney disease: an educational review. Pediatr Nephrol.

[33] Bower H, Bjorkholm M, Dickman PW, Hoglund M, Lambert PC, Andersson TM (2016). Life expectancy of patients with chronic myeloid leukemia approaches the life expectancy of the general population. J Clin Oncol.

[34] Capocaccia R, Gatta G, Dal Maso L (2015). Life expectancy of colon, breast, and testicular cancer patients: an analysis of US-SEER population-based data. Ann Oncol.

[35] Baade PD, Youlden DR, Andersson TM-L, Youl PH, Kimlin MG, Aitken JF (2015). Estimating the change in life expectancy after a diagnosis of cancer among the Australian population. BMJ Open.

[36] Bower H, Andersson TM, Bjorkholm M, Dickman PW, Lambert PC, Derolf AR (2016). Continued improvement in survival of acute myeloid leukemia patients: an application of the loss in expectation of life. Blood Cancer J.

[37] Gregg EW, Zhuo X, Cheng YJ, Albright AL, Narayan KM, Thompson TJ (2014). Trends in lifetime risk and years of life lost due to diabetes in the USA, 1985-2011: a modelling study. Lancet Diabetes Endocrinol.

[38] Gu K, Cowie CC, Harris MI (1998). Mortality in adults with and without diabetes in a national cohort of the US population, 1971-1993. Diabetes Care.

[39] Schneider H, Lischinski M, Jutzi E (1993). Survival time after onset of diabetes: 29-year follow-up mortality study in a diabetes cohort from a rural district. Diabete Metab.

[40] Rafati S, Baneshi MR, Hassani L, Bahrampour A (2019). Comparison of penalized cox regression methods in low-dimensional data with few-events: an application to dialysis patients' data. J Res Health Sci.

